# Correlation between Levels of Vitamins D_3_ and E in Type 2 Diabetes Mellitus: A Case-Control Study in Serdang, Selangor, Malaysia

**DOI:** 10.3390/nu13072288

**Published:** 2021-07-01

**Authors:** Nurliyana Najwa Md Razip, Banulata Gopalsamy, Mohd Sokhini Abdul Mutalib, Sui Kiat Chang, Muhammad Mikhail Joseph Anthony Abdullah, Azrina Azlan, Zulida Rejali, Huzwah Khaza’ai

**Affiliations:** 1 Department of Biomedical Sciences, Faculty of Medicine and Health Sciences, Universiti Putra Malaysia, Serdang 43400, Selangor, Malaysia; najwabiochem@gmail.com (N.N.M.R.); banulatagopalsamy@gmail.com (B.G.); 2 Department of Nutrition, Faculty of Medicine and Health Sciences, Universiti Putra Malaysia, Serdang 43400, Selangor, Malaysia; sukhini@upm.edu.my (M.S.A.M.); azrinaaz@upm.edu.my (A.A.); 3 Key Laboratory of Plant Resources Conservation and Sustainable Utilization, Guangdong Provincial Key Laboratory of Applied Botany, South China Botanical Garden, Chinese Academy of Sciences, Guangzhou 510650, China; changsk@scbg.ac.cn; 4 Department of Medicine, Faculty of Medicine and Health Sciences, Universiti Putra Malaysia, Serdang 43400, Selangor, Malaysia; avvai@yahoo.com; 5 Department of Obstetric and Gynaecology, Faculty of Medicine and Health Sciences, Universiti Putra Malaysia, Serdang 43400, Selangor, Malaysia; zulida@upm.edu.my

**Keywords:** diabetes mellitus, glycaemic status, insulin, lipid profiles, vitamin D_3_, vitamin E

## Abstract

An overview of vitamins D_3_ and E suggests micronutrient deficiency contributes to type 2 diabetes mellitus (T2DM). A case-control study was conducted to determine the status of plasma vitamins D_3_ and E isomers amongst diabetic Malaysians. Two groups were recruited for participation, one comprising fifty diabetic subjects (DM) and one comprising fifty non-diabetic (non-DM) subjects, in order to assess their plasma vitamin D_3_, calcium and vitamin E status. Glycaemic status (haemoglobin A1c, HbA1c; fasting blood glucose, FBG; C-Peptide) and lipid profiles (total cholesterol, TC; triglycerides, TG; low-density lipoprotein-cholesterol, LDL-C; high-density lipoprotein-cholesterol, HDL-C) were assessed, followed by anthropometric measurements. The Mann–Whitney U-test, Kruskal–Wallis and Spearman’s correlation coefficient were used to elucidate the association between levels of plasma vitamins D_3_ and E and T2DM. The vitamin D_3_ deficiency group (<20 ng/mL) showed a significant correlation (*p* < 0.05) with glycaemic status (HbA1c and FBG) and lipid profiles (HDL-C, LDL and TC). Spearman’s correlation demonstrated that vitamin D_3_ status is strongly correlated with HDL levels (*p* < 0.05). Similarly, plasma total vitamin E levels >4.9 μg/mL revealed significantly different FBG, HbA1c, C-Peptide, LDL, HDL and TC levels across both groups. Moreover, family history, smoking, waist circumference and HbA1c levels demonstrated a significant association (*p* < 0.05) with levels of vitamins D and E but not FBG and lipid profiles. This could be because the pre-diabetic status among the non-DM group influenced the outcomes of this study.

## 1. Introduction

Various recent studies have provided evidence of the association between deficiencies of plasma vitamins D_3_ [1,2,3] and E [4,5,6] and inflicting insulin resistance in type 2 diabetes mellitus (T2DM). Malaysia has the fifth-highest diabetes population in the Western Pacific region [7]. Furthermore, one in five Malaysians have been diagnosed with either pre-diabetes or diabetes, and a 4.9% increase in the diabetes population was recorded from 2015 to 2019. Comorbid diseases such as obesity, hyperlipidaemia, hypertension, cardiovascular disease and kidney disease are attributed to the pathophysiology of T2DM, which subsequently impacts economic sustainability.

Recent observational, cross-sectional, cohort and prospective studies have outlined risk factors associated with T2DM, mainly based on the nutrient status of individuals and how this affects the glycaemic status [8,9,10,11]. However, many research gaps remain unanswered in understanding the attributable risk factors that are associated with micronutrient status. Information on the status requirements of vitamins D_3_ and E would be beneficial in making long-term improvements to clinical guidelines and global health status.

Despite the superficial risk linked with the pathogenesis of T2DM, nutrition has emerged as the major underlying factor. Epidemiological evidence from various in vitro and in vivo studies indicate the importance of the effects of micronutrients, such as vitamins and minerals, in the T2DM development. Recent cross-sectional studies indicated associations between both vitamins D_3_ [12,13,14] and E [15] and insulin resistance. Nevertheless, the emerging evidence of the role of vitamins D and E in the development of T2DM suggests that further insight is required.

This present study was conducted to investigate the relationship between glycaemic status and deficiencies in levels of plasma vitamins D_3_ [16,17,18,19,20] and E [21,22]. Vitamins D_3_ and E are factors that can contribute to the pathophysiology of T2DM development. An optimum level of vitamin D in the body activates the vitamin D regulatory element, which is present in the pancreas and plays a role in restoring insulin sensitivity [20]. Vitamin E may modulate the action of beta-cells by scavenging free radicals and thus delaying T2DM progression [23]. Therefore, vitamins D and E have been cited as potential risk factors for T2DM based on studies of vitamin D [24,25] and vitamin E [23,26]. In addition, socio-demographic parameters (age, gender, ethnicity and education level), health status (smoking status and physical activity), medical background (family history of diabetes and predisposal to the disease) as well as biochemical measurements (glycaemic status and lipid profiles) have also been studied to determine how these factors may impact levels of vitamins D_3_ and E and to ascertain prominent indicators of the T2DM condition. The outcome of this study may provide a baseline reference for further intervention studies on micronutrients amongst diabetic patients in Malaysia.

## 2. Materials and Methods

### 2.1. Materials

Five millilitres of whole blood samples were collected into a vacutainer Test Grey-top KF^+^ Na_2_ EDTA (2 mL) and a BD Vacutainer Purple-top K_2_E (EDTA) (3 mL) (Becton Dickinson, Oxford, UK) and were centrifuged at 3000 rpm for 15 min at 4 °C to separate the red blood cells from the plasma. Then, 25 µL from the 5 mL whole blood were used in an HbA1c assay using the Direct Enzymatic HbA1c Assay kit (San Diego, CA, USA). Lipid profiles, including high-density lipoprotein cholesterol (HDL-C), low-density lipoprotein cholesterol (LDL-C), triglycerides (TG), total cholesterol (TC) and glycaemic status and serum fasting blood glucose (FBG) levels, were measured and analysed using a Roche Diagnostic bio-analyser; this was conducted in the medical diagnostic Haematology laboratory, Faculty of Medicine and Health Sciences, Universiti Putra Malaysia, Serdang, Selangor, Malaysia. C-peptide levels were measured using Enzyme-Linked Immunosorbent Assay Kit (Houston, TX, USA), according to the manufacturer’s instructions.

Standards of vitamin 1,25-dihydroxyvitamin D_3_ (calcitriol) and butylated hydroxytoluene (BHT) were purchased from Sigma-Aldrich (St. Louis, MO, USA). The standard of vitamin E isomers in the form of the tocotrienol-rich fraction (TRF) was kindly provided by Sime Darby Plantation Sdn. Bhd. HPLC grade methanol for analysis EMSUREACSISO was purchased from Merck KGaA (Lichrosolv, Darmstadt, Germany). Isopropanol and hexane were purchased from Friedemann Schmidt (USA).

### 2.2. Study Design and Subject Recruitment

This was a case-control study that involved patients aged 30 years and above who had been scheduled to visit the General Internal Medicine outpatient clinic, Serdang Hospital, Selangor, Malaysia. Recruitment was announced through advertisements. Interview sessions were conducted and consent to participate in this study was obtained prior to blood collection (Supplementary Material File S1). The diabetic subjects’ age ranges were matched with those of healthy subjects and selected based on the medical records held in Serdang Hospital. To qualify for selection, the subjects’ FBG levels needed to be >6.7 mmol/L and the HbA1c levels needed to be >6.5%, according to the guidelines outlined by the American Diabetes Association [27]. The selection of non-DM and DM participants was based on inclusion and exclusion criteria. Inclusion criteria were adults aged between 30 and 65 years old who had received outpatient treatment in Serdang Hospital. Exclusion criteria were any diabetic patients and non-diabetic cases with nephropathy complications, oral contraceptive consumers, pregnant women, thyroid and parathyroid cases, those with bone diseases or cancers and subjects who were consuming vitamin D_3_, calcium or Omega-3 fatty acids supplements. Meanwhile, the selection of non-DM subjects was made based on self-reporting on their capacity as healthy, according to interview sessions held one day before the blood sample collection.

### 2.3. Sample Size Calculation

Sample size of the subjects was calculated based on the formula of statistical software package G*Power 3.17 using two-independent means (two groups) formula. The number of subjects recruited, was estimated from 7.8% T2DM prevalence in the Malaysian National Health Morbidity Survey in 2015 for the area in Serdang, Selangor, [28]. Thus, the sample size required was 100 subjects’ recruitment with 50 subjects of DM and 50 subjects of non-DM groups.

### 2.4. Socio-Demographic Parameters

Socio-demographic information, which included age, gender, ethnicity, smoking status, physical activity level, education level and marital status, were obtained through interview sessions with the subjects using self-administered questionnaires.

### 2.5. Anthropometric Assessment

The heights in (m) of the subjects were measured using the SECA stadiometer Model 220 (SECA, Hamburg, Germany) scale to the nearest 0.1 cm. Weight in (kg) was measured using the SECA-Biometric electronic scale (Tanita Health Equipment Ltd., Tokyo, Japan) to the nearest 0.1 kg. Body mass index (BMI) (weight (kg)/height^2^ (m^2^)) was calculated based on the formula recommended by the World Health Organization (WHO) [29]. BMI cut-off in Asian adults is categorised as underweight (BMI < 18.5 kg·m^−2^), normal (BMI 18.5–24.9 kg·m^−2^); overweight (BMI > 25 kg·m^−2^) and obese (BMI > 30 kg·m^−2^), according to WHO guidelines [30]. Waist circumference was measured using a measuring tape held at the point of the minimal waist. The measurement was compared with cut-off values according to WHO guidelines [30], whereby males with a waist circumference >90 cm and female with waist circumference >80 cm were indicated as centrally obese. All measurements were performed in triplicate (*n* = 3) and the mean ± SD values were used for data analysis.

### 2.6. Direct Enzymatic HbA1c Assay

The HbA1c test measures the thickness of the glucose coating of the red blood cells over the past three months. The threshold of normal HbA1c is less than 6.5% according to the American Diabetes Association [27]. Twenty-five microlitres of whole blood were allocated for the measurement of HbA1c. The procedures for conducting the HbA1c assay were based on the manufacturer’s protocol. The signal produced by the reaction was used to report the HbA1c (%) of the sample using a linear calibration curve.

### 2.7. C-Peptide Enzyme-Linked Immunosorbent Assay Kit

The C-Peptide level is a more effective biomarker for insulin as it represents endogenous insulin. Based on the manufacturer’s protocol, 50 μL of serum were utilised in the competitive inhibition enzyme assay.

### 2.8. Vitamin D_3_ Analysis Using High-Performance Liquid Chromatography (HPLC)

#### 2.8.1. Extraction of Vitamin D_3_ from Blood Plasma

The plasma vitamin D_3_ level was extracted and determined according to Turpeinen et al. [31] with minor modifications. Furthermore, 500 μL of plasma sample were added to 350 μL of HPLC grade methanol (Merck, Darmstadt, Germany) and isopropanol (Friendemann Schmidt, Parkwood, WA, Australia) in the ratio of 80:20 (*v*/*v*). The solvent mixture was vortexed for 30 s. Two mL of HPLC grade *n*-hexane (Friendemann Schmidt, Parkwood, WA, Australia) were added to the mixture and vortexed for 60 s. After that, the supernatant and a pellet of the sample were separated by centrifugation (3000 rpm), in which the upper organic phase was transferred into a conical tube. The extraction was repeated thrice for each sample and finally the solvent mixture was dried under nitrogen gas. Then, the residue was dissolved in 100 µL of water and methanol in the ratio of 76:4 (*v*/*v*). The sample was filtered using a non-sterile polyvinyl difluoride (PVDF) filter (pore size = 0.22 µm and diameter 13 mm) (AMTEC Sdn.Bhd, Klang, Selangor, Malaysia) and the eluent was collected in a 1.5 mL amber vial. The calibration curve was plotted using six different concentrations of vitamin D_3_ (3.125, 6.25, 12.5, 25, 50 and 100 ng/mL) to form the reference standard.

#### 2.8.2. Analysis of the Level of Plasma Vitamin D_3_

The plasma vitamin D_3_ concentration was measured using the HPLC Agilent 1100 system (Agilent Technologies, Waldbronn, Germany) connected to a reverse-phase Kinetex 5 µm C18 100A column (250 mm × 4.6 mm; Phenomenex Inc., Torrance, CA, USA) with a quaternary pump solvent delivery system and a UV detector maintained at 40 °C. The mobile phase used was acetonitrile and methanol in the ratio of 88:12 (*v*/*v*), using an isocratic gradient. The injection volume was 20 µL with a flow rate of 2.0 mL/min, with UV detection at 265 nm. Data interpretation was made using the Chemstation Agilent Operation System (Agilent Technologies, Waldbronn, Germany). The amount of vitamin D_3_ in each subject’s plasma sample was validated using standard curve vitamin D_3_ (cholecalciferol).

### 2.9. Analysis of Plasma Total Vitamin E and Its Isomers

The vitamin E extraction method was adapted from Matyash et al. [32] with minor modifications. To avoid oxidation during the extraction, vitamin E was extracted by adding 200 µL of plasma with 1.5 mL 0.1% butylated hydroxytoluene (BHT); this was vortexed. Then, 5 mL of methyl-tert-butyl-ether (MTBE) was added and the mixture was incubated for 1 h at room temperature. Phase separation was induced by adding 1.25 mL of ultrapure water. The mixture was incubated for 10 min at room temperature and was centrifuged at 3000 rpm. The upper organic phase was collected, and the lower phase was re-extracted with 2 mL of the solvent mixture containing MTBE/methanol/water 10:3:2.5 (*v*/*v*/*v*). Samples with combined organic phases were dried and concentrated under vacuum. To accelerate the sample drying process, 200 µL of hexane was added to the organic phase after 25 min of centrifugation (3000 rpm). The sample was then reconstituted with mobile phase, hexane and isopropanol in the ratio of 99:1 (*v*/*v*) before further analysis was conducted using HPLC. The denotation of vitamin E isomers was analysed using the HPLC Agilent 1100 equipped with a quaternary pump. The column was a YMC-pack silica, 150 × 6.0 mm, 5 µm column operated at 40 °C. The mobile phase was hexane and isopropanol in the ratio of 99:1 (*v*/*v*), with a flow rate of 1.5 mL/min. The settings of the fluorescent detector were E_x_, excitation wavelength at 295 nm and E_m_, emission wavelength at 325 nm. All the chromatograms’ data were analysed using the ChemStation Operation System (Agilent Technologies, Waldbronn, Germany).

### 2.10. Statistical Analysis

The Statistical Package for Social Sciences version 22.0 (SPSS 22.0, Inc.Chicago, IL, USA) was used for statistical analysis in this study. Data skewness and kurtosis were used to determine the normality of all data sets. Data were expressed as the mean ± SD or SEM or percentage (%) for the categorical data. Comparisons between the two groups were performed using the independent samples t-test or Mann–Whitney U test (non-parametric continuous variables) and one-way ANOVA or Kruskal–Wallis test. The vitamin D_3_ status was stratified into three groups based on the recommendation of the Institute of Medicine (IOM): optimal (20–80 ng/mL), deficiency (<20 ng/mL) and toxicity (>80 ng/mL) [33]. Spearman’s correlation coefficient was used to determine the association between vitamin D_3_, vitamin E and calcium in diabetic cases. In this study, the determination of the correlation study was performed following the significant *p*-value, at the levels of either <0.05 or <0.001 in a 2-tailed analysis. Multivariate analysis was used to validate the confounding relative risk factors. All factors were analysed through univariate logistic regression in the multivariate analysis to obtain the odds ratio (OR) and confidence interval (CI, 95%).

## 3. Results

### 3.1. Descriptive Characteristics

The socio-demographic and anthropometric characteristics of 50 diabetic (DM) and 50 non-diabetic (non-DM) subjects are presented in Table 1. There were almost equal numbers of male and female subjects in both groups. The majority of the subjects were Malays and married. The data showed that 90% of the non-DM subjects and 86% of the DM subjects had attained tertiary level education. The waist circumference (95.12 ± 13.54 for the non-DM and 103.4 ± 16.17 cm for the non-DM subjects) and body weight (73.68 ± 15.8 kg for non-DM and 78.5 ± 15.14 kg for DM subjects) were not significantly different (*p* > 0.05) between both groups. The average heights were 163.8 ± 7.9 and 164.4 ± 8.67 cm for the subjects in the non-DM and DM groups, respectively. In the non-DM group, 28% of the subjects were obese, while 43% of the subjects of the DM group fell into the obese category. However, no statistically significant difference (*p* > 0.05) was observed between both groups. Meanwhile, the levels of plasma vitamins D and total vitamin E showed no significant difference (*p* > 0.05) when the gender, age and ethnicity categories were examined, nor when comparing between the non-DM and DM groups.

The medical history of the subjects (as shown in Table 2) showed that 62% of the DM subjects had a family history of diabetes while 46% of non-DM subjects’ family members had diabetes. Those in the DM group also reported other complications such as cardiovascular disease (12%), gastrointestinal disease (16%), hyperlipidaemia (34%), renal disease (14%), arthritis (28%) and epilepsy (2%). A total of 14% of the subjects did not provide an answer to this question.

Lifestyle behaviour such as smoking habits and the physical activity of the subjects (shown in Table 3) showed no significant difference (*p* > 0.05) between the groups. Seventy-eight per cent of the subjects from the non-DM group and 84% of the DM group had never smoked. More than 50% of the subjects also recorded physical activity status that fell into the ‘good category’. With these parameters, all the factors were analysed to determine significance using a base frequency of 3–5 times per week.

### 3.2. The Association of Levels of Plasma Vitamin D_3_, Calcium and Vitamin E in Connection with Glycaemic Status and Lipid Profiles

To elucidate the relationship between the vitamin D_3_ status and T2DM, the glycaemic status and lipid profiles were assessed in this study. Table 4 shows that a major percentage of the subjects in both the non-DM (82%) and DM (84%) groups were vitamin D_3_ deficient. The outcome of the calcium status assessment indicated that subjects from the DM group recorded significantly higher (*p <* 0.05) calcium levels (>10.3 mg/dL) when compared to those in the non-DM group. However, more than 50% of the subjects in both groups recorded low calcium levels. The glycaemic status of the subjects in the non-DM groups fell within the range outlined by the subjects’ FBG levels of <6.7 mmol/L and HbA1c of <6.5%, according to guidelines outlined by the American Diabetes Association [27]. To determine the association between plasma vitamin D_3_ and biochemical parameters, the main focus was the vitamin D_3_ deficient category (<20 ng/mL) and the glycaemic status in non-DM and DM groups was assessed. The findings indicate a strong association between vitamin D_3_ and glycaemic status; HbA1c (9.45 ± 2.51%) and FBG (9.48 ± 3.19 mmol/L) were significantly higher (*p* < 0.05) in the DM group. For the lipid profiles, TC (4.18 ± 1.40 mmol/L), LDL-C (2.14 ± 0.58 mmol/L) and HDL-C (1.08 ± 0.60 mmol/L) were significantly higher (*p* < 0.05) in the non-DM group. In order to confirm whether calcium works synergistically with vitamin D_3_, the low plasma calcium level along with the glycaemic status and lipid profiles were also measured (see Table 4). Low plasma calcium levels (<8.6 mg/dL), with HbA1c (9.24 ± 2.49%), FBG (8.94 ± 3.31 mmol/L) and C-Peptide (2.19 ± 0.24 ng/mL), were significantly higher (*p* < 0.05) in the DM group. Similarly, LDL-C (2.38 ± 0.58 mmol/L) and HDL-C (1.42 ± 0.60 mmol/L) were also significantly higher (*p* < 0.05) in the non-DM group.

To identify risk factors associated with vitamin D_3_ deficiency, multiple logistic regression was performed, in which all the factors were analysed to determine the significance using univariate logistic regression in multivariate analysis to obtain the odds ratio (OR) and confidence interval (95% CI) corresponding to *p* < 0.05. To identify risk factors for vitamin D_3_ deficiency, a new variable was used based on the percentage agreement of plasma vitamin D_3_ levels, as shown in Table 5. All the subjects with vitamin D_3_ deficiency (<20 ng/mL) were classified to be at risk of T2DM including those non-DM subjects with higher HbA1c levels. Some participants who did not meet the categorisation criteria were considered participants with no risk factors for vitamin D_3_ deficiency. Binary variables were used to identify risk factors associated with vitamin D_3_ deficiency using a logistic regression test. Table 6 shows that a number of risk factors may increase the probability of vitamin D_3_ deficiency; therefore, most variables were added as independent covariates, while the indicator of vitamin D_3_ deficiency was the outcome. The model adopted the continuous and categorical variables listed below:

i. Smoking status (current smoker and ex-smoker)
ii. Physical activity (good)
iii. Educational level (secondary and tertiary)
iv. Marital status (single)
v. Family history (of diabetes)
vi. HDL (male and female)
vii. LDL (>2.6 mmol/L)
viii. TC (>5.2 mmol/L)
ix. FBG (>6.61 mmol/L)
x. HbA1c (>6.5%)
xi. C-peptide (>3.31 ng/mL)
xii. Calcium (<8.6 mg/dL and no deficiency of calcium)

The overall status of the model was constant with a significance value of 0.23. The Nagelkerke R square equals 0.23, indicating that 23% of the variation of the outcome was vitamin D_3_ deficiency. Wald statistics were used to explain the importance of the contribution of each variable in the model. Results from this study show that the multivariate analysis model did not indicate any relative risk, according to the odds ratio value. The *p*-values were all above 0.05, indicating there was no significant difference between vitamin D_3_ deficiency in the subjects and the risk factors.

The vitamin E standard from the extracted palm oil contained five isomers. As shown in Table 7, the levels of plasma total vitamin E were not significantly different (*p* > 0.05) across the two groups. The DM group exhibited 18% higher plasma total vitamin E (4.9 ± 4.3 µg/mL) than non-DM (3.97 ± 3.33 µg/mL). Plasma α-tocopherol, α-tocotrienol, β-tocotrienol and δ-tocotrienol levels were not significantly different (*p* > 0.05) across both groups. Plasma γ-tocotrienol in the DM (1.13 ± 0.20 µg/mL) subjects was significantly higher (*p* < 0.05) than for those in non-DM (0.65 ± 0.23 µg/mL) group.

Table 8 illustrates the relationship between levels of plasma total vitamin E and the biochemical parameters in the two groups. The plasma total vitamin E level was classified into two categories: <4.9 µg/mL or >4.9 µg/mL. Subjects with plasma total vitamin E levels of <4.9 µg/mL revealed significant differences (*p* < 0.05) in FBG, HbA1c, LDL-C and TC levels between the DM and non-DM groups. For the subjects with >4.9 µg/mL of plasma total vitamin E, all three glycaemic parameters (FBG, HbA1c, C- peptide) and the LDL-C, HDL-C and TC levels were significantly higher (*p* < 0.05) in the subjects in the DM group compared to those in the non-DM group.

The univariate analysis was corroborated with multivariate analysis of plasma total vitamin E levels to enable the dependent cofounding factors to be predicted. Table 9 shows that a population’s risk factors may increase the probability of a vitamin E concentration of <4.9 mg/mL when most variables are added as independent covariates from the risk factors of the vitamin E status baseline are shown in Table 10. A selection of covariates was made from the univariate and bivariate analyses to describe the potential association with vitamin E status. The model adopted continuous and categorical variables. The continuous variables were age, waist circumference and BMI. Meanwhile, the categorical variables are listed below:

i. Smoking status (current smoker and ex-smoker)
ii. Physical activity (good)
iii. Educational level (lower secondary, secondary and tertiary)
iv. Marital status (single)
v. Family history (of diabetes)
vi. HDL (male and female)
vii. LDL (>2.6 mmol/L)
viii. TC (>5.2 mmol/L)
ix. FBG (>6.61 mmol/L)
x. HbA1c (>6.5%)
xi. C-peptide (>3.31 ng/mL)

The overall status of the model was not significant, as the constant significant value was 0.25. The Nagelkerke R square equals 0.28, indicating that 28% of the subjects with total vitamin E levels of >4.9 µg/mL have multiple risk factors. The data of the multivariate analysis model showed relative levels of risk, according to the results of the odds ratio. The value was significant at *p* < 0.05. Generally, waist circumference (OR = 1.055, *p*-value = 0.02, 95% CI = 1.010–1.102) and FBG (OR = 3.21, *p*-value = 0.05, 95% CI = 0.98–10.47) were identified as significant risk factors for high vitamin E levels to scavenge free radicals in T2DM pathophysiology.

The correlation from the case-control study demonstrated the association of the independent variables, mainly the levels of plasma vitamin D_3_, calcium, α-tocopherol and total vitamin E, with the factors listed in Table 11. Heat maps of the bivariate analysis for the Spearman correlation rho value (r_s_) (Table 11) exhibited positive correlations among the non-DM group, showing that age (r_s_ = 0.30) and waist circumference (r_s_ = 0.40) were associated with plasma total vitamin E and α-tocopherol levels, respectively. Meanwhile, C-Peptide (r_s_ = 0.35) was positively correlated with calcium but HDL (r_s_ = −0.34) and total vitamin E levels (r_s_ = −0.34) were negatively correlated with calcium levels. On the other hand, in the DM group, a positive association was found between calcium levels and family history of diabetes (r_s_ = 0.40), smoking status (r_s_ = 0.37) and waist circumference (r_s_ = 0.39). Plasma total vitamin E levels showed a positive correlation with smoking status (r_s_ = 0.41). HbA1c levels demonstrated a positive association with levels of plasma α-tocopherol (r_s_ = 0.38) and total vitamin E (r_s_ = 0.41). HDL-C levels (r_s_ = 0.30) showed a positive correlation with plasma vitamin D_3_. While calcium showed a negative correlation with HDL-C (r_s_ = −0.36), LDL-C (r_s_ = −0.32), TC (r_s_ = −0.39) and TG (r_s_ = −0.37) levels.

## 4. Discussions

This study has limitations as a case-control designation and a convenience sampling which indicate an imbalanced disparity between age, gender and ethnicity. Although the main focus in this study was the association of the status of vitamins D and E in T2DM development, herein showed the pre-diabetic factors in the non-DM group. This preliminary data revealed the prevalence of vitamin D deficiency in more than 80% of subjects. Besides, with deficiency in vitamin D, vitamin E was also found to be low in 56% of subjects in the DM and non-DM groups.

The vitamin D deficient category was the focus in this study, as was the glycaemic status and lipid profiles. Along with this, low calcium plasma levels showed similar results to the vitamin D deficient category in the DM and non-DM groups. Their plasma calcium levels were also a matter of concern, since more than 50% of the control and diabetic groups showed low calcium levels. This can be corroborated by Zhao et al. [34], who determined that vitamin D deficiency has a relationship with T2DM progression. In addition, the glycaemic status showed as significantly higher in the DM group, which in the vitamin D deficient category aligned with results found by Kumar et al. [2] and Alemzadeh et al. [35]. Therefore, this highlights the possibility that vitamin D_3_ levels might be a good indicator for diabetes since low vitamin D_3_ levels may cause insulin resistance and impaired *β*-cell function in the pancreas [36,37,38]. The results from this study indicated that the high glycaemic status in the DM group was found to be due to deficiencies in vitamin D and calcium. This can be corroborated by previous findings by Fong et al. [39], Pittas et al. [40] and Jan Mohamed et al. [41] who demonstrated that calcium works synergistically with vitamin D in restoring insulin sensitivity. Thus, the data obtained can be considered a primary outcome to substantiate the importance of vitamin D_3_ status in DM subjects.

On the other hand, total vitamin E deficient status in this study was found to be a concern in the diabetes cases which revealed 56% of the subjects with less than 4.9 μg/mL. Commonly, the optimum range of total vitamin E levels reported in adolescents varies between 3–15 µg/mL [42] and 5.5–17 µg/mL [43], as the findings of observational studies indicate. Total vitamin E includes isomers of α-tocopherol, α-tocotrienol, β-tocotrienol, γ-tocotrienol and δ-tocotrienol. The α-tocotrienol levels were higher than the α-tocopherol levels in the DM subjects. The α-tocotrienol was the major tocotrienol isomer detected in the plasma and lipoprotein fractions [4]. Similarly, the absorption of α-tocotrienol was shown to be substantially higher than the absorption of γ-and δ-tocotrienols [44,45]. In line with the results of a study by Vatassery et al. [46], this study reported higher total vitamin E levels in DM subjects, whereas Khabaz et al. [47] suggested that α-tocopherol levels were found to be low in diabetic patients.

Nevertheless, the association of diabetes cases based on either FBG or HbA1c levels with vitamin D_3_ status was a negligible factor in the Spearman’s correlation study. Although vitamin D_3_ deficiency showed no association with the DM group, but, total vitamin E and α-tocopherol were strikingly exhibited to be associated with T2DM progression based on the HbA1c values. This is aligned with previous studies reporting the relationship between vitamin E status and the incidence of T2DM [15,48,49,50,51].

Apart from that, the lipid profile status indicated the association of HDL-C, LDL-C, TG and TC with calcium but not with vitamin D_3_. Theoretically, activated vitamin D_3_ has lipid-lowering effects according to Drincic et al. [52] and Hengist et al. [53], through a cooperation between the calcium and vitamin D in mechanistic action. Similarly, with vitamin D_3_, vitamin E and α-tocopherol did not show association with lipid profiles.

Additionally, socio-demographic factors could be the contributors to vitamin D deficiency. Most of the subjects aged over 35 years old were categorised as vitamin D_3_ deficient [54,55], however the age factor did not indicate an association with vitamin D_3_ status. In contrast, the older age group, with high BMI and waist circumference measurements, was shown to have a strong association with vitamin D status by Shafinaz and Moy [54] and Tavakoli et al. [56]. Vitamin E status coincides with the age factor, indicating a contributing factor to diabetes, which is aligned with a previous study [57]. Besides, Ismail et al. [58] demonstrated that smoking status has an association with total vitamin E, which is similar to the current study findings.

The outcomes of this study indicate that the micronutrients, vitamins D_3_ and E, are indispensable for a better improvement of glycaemic status and lipid profiles amongst the diabetic and pre-diabetics subjects.

## 5. Conclusions

To conclude, this case-control study assessed pre-diabetes and diabetes with high prevalence of vitamins D and E deficiencies. High HbA1c levels are associated with α-tocopherol and total vitamin E, but not with vitamin D_3_ and calcium. This may highlight the importance of maintaining sufficient plasma levels of vitamins D and E in curbing the progression of T2DM development.

## Figures and Tables

**Table 1:** Socio-demographic characteristics and anthropometric assessment of subjects in non-DM and DM groups.

Variables	Group		*p*-Value < 0.05	Vitamin D_3_ (ng/mL)	Total Vitamin E (μg/mL)
	Non-DM, *n* = 50 (%)	DM, *n* = 50 (%)			
	Gender				
Male	28 (56)	26 (51)	-	11.94 ± 25.96	2.17 ± 4.54
Female	22 (44)	24 (48)	-	15.41 ± 31.29	1.32 ± 2.30
			*p*-value	0.55	0.25
	Age (years)				
≤35	11 (22)	3 (6)	-	4.90 ± 5.36	3.63 ± 3.75
>35–50	21 (42)	16 (32)	-	10.58 ± 19.20	5.03 ± 4.31
>50–65	18 (36)	27 (54)	-	19.51 ± 37.84	4.42 ± 3.54
>65	-	4 (8)	-	3.57 ± 6.18	1.35 ± 0.38
			*p*-value	0.68	0.10
	Ethnicity				
Malay	45 (90)	33 (66)	-	12.47 ± 26.27	4.59 ± 3.64
Chinese	1 (2)	9 (18)	-	8.46 ± 11.07	2.67 ± 4.09
Indian	4 (8)	8 (16)	-	24.70 ± 46.73	4.93 ± 4.92
			*p*-value	0.80	0.10
	Marital status				
Married	45 (90)	46 (92)	-	-	-
Divorced	4 (8)	2 (4)	-	-	-
Single	1 (2)	2 (4)	-	-	-
	Education level				
Lower	-	-	-	-	-
Secondary	5 (10)	7 (13)	-	-	-
Tertiary	45 (90)	43 (86)	-	-	-
	Height (cm)				
Mean ± SD	163.8 ± 7.91	164.4 ± 8.67	-	-	-
	Waist circumference (cm)				
Mean ± SD	95.12 ± 13.54	103.4 ± 16.17	<0.05	-	-
	Body weight (kg)				
Mean ± SD	73.68 ± 15.81	78.50 ± 15.14	0.36	-	-
	Body mass index (BMI) (kg/m^2^)				
Normal (18.5–24.9)	13 (26)	8 (16)	0.09	-	-
Overweight (≥25)	23 (46)	20 (40)		-	-
Obesity (≥30)	14 (28)	22 (44)		-	-
Mean ± SD	27.31 ± 4.63	28.69 ± 4.80		-	-
Vitamin D_3_ (ng/mL)	11.73 ± 20.49	15.35 ± 34.76	0.25	-	-
Total vitamin E (µg/mL)	3.97 ± 3.33	4.9 ± 4.3	0.09	-	-

Non-DM: non-Diabetes Mellitus, DM: Diabetes Mellitus. All data were expressed in either number (percentage) or mean ± standard deviation (SD). The categorical variables were expressed in descriptive statistics. Significant level was set at *p* < 0.05.

**Table 2:** Medical history of subjects in non-DM and DM groups.

Medical History	Non-DM *n* = 50 (%)	DM *n* = 50 (%)
Family history of diabetes		
Yes	23 (46)	31 (62)
No	27 (54)	19 (38)
Subjects with other comorbid diseases		
Cardiovascular disease (CVD)	-	6 (12)
Gastrointestinal disease (GI)	4 (8)	8(16)
Hyperlipidaemia	4 (8)	17 (34)
Renal disease (RD)	-	7 (14)
Arthritis	3 (6)	14 (28)
Epilepsy	-	1 (2)
Missing	39 (78)	7 (14)

Statistical analysis was expressed in percentage (%).

**Table 3:** Lifestyle behaviour of subjects in non-DM and DM groups.

Lifestyle Behaviour	Group		Chi Square Test	
	Non-DM *n* = 50 (%)	DM *n* = 50 (%)	Value	*p*-Value
Smoking habits				
Current smokers	7 (14)	5 (10)	0.59	0.75
Ex-smokers	4 (8)	3 (6)		
Never smoked	39 (78)	42 (84)		
Physical activity				
Good (3–5 times per week)	27 (54)	27 (54)	1.89	0.60
Fair (<3 times per week)	13 (26)	17 (34)		
Poor (never exercise)	10 (20)	6 (12)		

Crosstabs test was adopted in this statistical analysis. Significant level was set at *p* < 0.05.

**Table 4:** Distribution of plasma vitamin D_3_ and calcium levels in non-DM and DM groups determined using Independent T-test.

Variables	Non-DM Mean ± SD, *n* = 50 (%)	DM Mean ± SD, *n* = 50 (%)	Test Value	*p*-Value
Vitamin D_3_ status				
Deficiency (<20 ng/mL)	3.43 ± 2.65, (82%)	4.437 ± 2.86, (84%)	Z = −1.345	ns
Optimal (20–80 ng/mL)	33.79 ± 20.41, (16%)	37.88 ± 21.08, (10%)	Z = −0.732	ns
Toxicity (>80 ng/mL)	113.5 ± 25.19, (2%)	141.7 ± 26.58, (6%)	Z = −1.342	ns
Vitamin D_3_ deficiency (<20 ng/mL): Glycaemic status				
HbA1c (%)	6.67 ± 1.97	9.45 ± 2.51	Z = 5.00	<0.05 *
FBG (mmol/L)	5.57 ± 0.72	9.48 ± 3.19	Z = −6.19	<0.05 *
C-Peptide (ng/mL)	2.11 ± 0.35	2.23 ± 0.29	Z = −1.40	ns
Vitamin D_3_ deficiency (< 20 ng/mL): Lipid profiles				
TG (mmol/L)	1.12 ± 0.57	1.05 ± 0.43	Z = −0.11	ns
TC (mmol/L)	4.18 ± 1.40	3.22 ± 1.42	Z = −3.14	<0.05 *
LDL-C (mmol/L)	2.14 ± 0.58	1.47 ± 0.81	Z = −4.58	<0.05 *
HDL-C (mmol/L)	1.08 ± 0.60	0.66 ± 0.48	Z = −3.05	<0.05 *
Calcium status				
Low (<8.6 mg/dL)	5.3 ± 2.7, (56%)	5.8 ± 1.49, (50%)	Z = −0.23	ns
Normal (8.6–10.3 mg/dL)	9.4 ± 1.4, (10%)	9.6 ± 2.99, (9%)	Z = −0.60	ns
High (>10.3 mg/dL)	12.2 ± 2.95, (34%)	14.6 ± 2.15, (44%)	Z = −3.22	<0.05 *
Low calcium (< 8.6 mg/dL): Glycaemic status				
HbA1c (%)	7.07 ± 1.59	9.24 ± 2.49	Z = −3.62	<0.05 *
FBG (mmol/L)	5.37 ± 0.53	8.94 ± 3.31	Z = −4.87	<0.05 *
C-Peptide (ng/mL)	2.02 ± 0.31	2.19 ± 0.24	Z = −2.01	<0.05 *
Low calcium (< 8.6 mg/dL): Lipid profiles				
TG (mmol/L)	1.22 ± 0.57	1.24 ± 0.48	Z = −0.25	ns
TC (mmol/L)	4.41 ± 0.85	3.81 ± 1.26	Z = −1.83	ns
LDL-C (mmol/L)	2.38 ± 0.58	1.76 ± 0.79	Z = −3.27	<0.05 *
HDL-C (mmol/L)	1.42 ± 0.60	1.00 ± 0.69	Z = −2.00	<0.05 *

HbA1c: Haemoglobin A1c, FBG: Fasting Blood Glucose, TG: Triglycerides, TC: Total Cholesterol, LDL-C: Low Density Lipoprotein Cholesterol, HDL-C: High Density Lipoprotein Cholesterol. Significant level was set at *p* < 0.05 (*). Mann–Whitney U-test (non-parametric test) was used in the statistical analysis.

**Table 5:** Multivariate analysis of vitamin D_3_ deficiency.

New Variables	SPSS Coding	Participants	N (%)
No deficiency	0	8	8
Deficiency of vitamin D_3_ (< 20 ng/mL)	1	92	92

**Table 6:** Prediction of risk factors associated with vitamin D_3_ deficiency.

Covariates	S.E	Wald	Sig.	OR	95% CI for OR	
					Lower	Upper
Age	0.04	0.49	0.48	0.98	0.91	1.05
Waist (cm)	0.03	0.05	0.82	0.99	0.95	1.04
BMI (kg/m^2^)	0.09	0.09	0.76	1.03	0.87	1.21
Smoking status		0.46	0.79			
Current smoker	0.86	0.40	0.53	0.58	0.11	3.13
Ex-smoker	1.40	0.03	0.86	1.28	0.08	19.94
Physical activity (good)	0.96	0.08	0.78	0.76	0.12	4.97
Education level	1.41	0.65	0.42	3.13	0.20	49.59
Marital status (single)	1.02	0.72	0.40	0.42	0.06	3.09
Family history	0.70	0.01	0.92	1.07	0.27	4.21
HDL (male)	0.84	1.08	0.30	2.38	0.46	12.28
HDL (female)	0.98	0.29	0.59	0.59	0.09	4.03
LDL (>2.6 mmol/L)	0.89	0.83	0.36	2.25	0.40	12.78
TC (>5.2 mmol/L)	1.02	0.03	0.87	0.84	0.11	6.20
FBG (>6.61 mmol/L)	0.80	0.02	0.90	1.11	0.23	5.27
HbA1c (>6.5%)	0.76	1.57	0.21	0.39	0.09	1.71
C-peptide (>3.1 ng/mL)	1.13	1.08	0.30	0.31	0.03	2.83
Calcium (mg/dL)	0.74	1.81	0.18	2.69	0.64	11.38
Constant	4.50	1.22	0.27	144.02		

Reference category. OR = odds ratio. S.E = standard error. CI = confidence interval. Association significant was set at *p* < 0.05 level (2-tailed). Multiple logistic regression was adopted in this analysis.

**Table 7:** Plasma vitamin E isomer levels in non-DM and DM groups.

Isomers of Vitamin E (µg/Ml)	Mean ± SD		Test Value	*p*-Value
	Non-DM, *n* = 50	DM, *n* = 50		
α-Tocopherol	2.18 ± 1.98	2.11 ± 1.54	t = 0.19	0.17
α-Tocotrienol	0.94 ± 0.18	1.39 ± 0.55	t = −1.61	0.22
β-Tocotrienol	0.01 ± 0.02	0.01 ± 0.03	Z = −0.63	0.53
γ-Tocotrienol	0.65 ± 0.23	1.13 ± 0.20	t = −2.13	<0.05 *
δ-Tocotrienol	0.12 ± 0.06	0.18 ± 0.05	t = −1.31	0.16
Total vitamin E	3.97 ± 3.33	4.90 ± 4.30	t = −1.21	0.09

Significant level was set at *p* < 0.05 (*). Independent sample t-test and Mann–Whitney U-test were adopted for the parametric and non-parametric test, respectively.

**Table 8:** The relationship of plasma total vitamin E with biochemical parameters in groups using the cut-off baseline adopted from plasma total vitamin E in both groups.

Variables	Total vitamin E							
	<4.9 µg/mL, *n* = 56		Test Value	*p*-Value	>4.9 µg/mL, *n* = 44		Test Value	*p*-Value
	Non-DM, *n* = 29	DM, *n* = 27			Non-DM, *n* = 21	DM, *n* = 23		
*Glycaemic status*								
FBG (mmol/L)	5.75 ± 0.80	9.16 ± 2.92	t = −6.05	<0.05 *	5.33 ± 0.51	9.36 ± 3.56	t = −5.36	<0.05 *
HbA1c (%)	6.80 ± 2.23	8.58 ± 2.53	t = −2.79	<0.05 *	6.71 ± 1.70	10.19 ± 2.49	t = −5.36	<0.05 *
C-peptide (ng/mL)	2.14 ± 0.31	2.21 ± 0.28	t = −0.89	0.38	2.05 ± 0.38	2.26 ± 0.29	t = −2.05	<0.05 *
*Lipid profile status*								
LDL-C (mmol/L)	2.12 ± 0.53	1.38 ± 0.72	t = 4.40	<0.05 *	2.38 ± 0.65	1.57 ± 0.83	t = 3.57	<0.05 *
HDL-C (mmol/L)	1.04 ± 0.67	0.80 ± 0.52	t = 1.51	0.14	1.27 ± 0.58	0.77 ± 0.65	t = 2.68	<0.05 *
TC (mmol/L)	4.13 ± 1.30	3.12 ± 1.36	t = 2.83	<0.05 *	4.59 ± 1.38	3.38 ± 1.37	t = 2.91	<0.05 *
TG (mmol/L)	1.17 ± 0.62	1.09 ± 0.53	t = 0.48	0.63	1.16 ± 0.54	1.07 ± 0.37	t = 0.66	0.51
*Other profile status*								
Vitamin D_3_ (ng/mL)	12.32 ± 21.44	15.94 ± 29.58	t = −0.53	0.60	10.09 ± 15.27	8.44 ± 14.60	t = 0.37	0.72
Calcium (mg/dL)	8.33 ± 3.84	9.04 ± 4.78	t = −0.61	0.54	7.79 ± 3.67	10.18 ± 4.32	t = −1.97	0.06

Significant level was set at *p* < 0.05 (*). Independent t-test was adopted in the data analysis.

**Table 9:** Multivariate analysis of plasma vitamin E level.

New Variables	SPSS Coding	Participants	*n* (%)
<4.9 µg/mL	0	56	56
>4.9 µg/mL	1	44	44

**Table 10:** Prediction of risk factors associated with plasma vitamin E level.

Covariates	S.E	Wald	Sig.	OR	95% CI for OR	
					Lower	Upper
Age	0.03	0.04	0.85	0.10	0.95	1.05
Waist Circumference (cm)	0.02	5.87	0.02*	1.06	1.01	1.10
BMI	0.06	0.32	0.58	0.96	0.85	1.09
Smoking status		2.64	0.27	0.57	0.11	2.92
Current smoker	0.84	0.46	0.50	0.57	0.11	2.92
Ex-smoker	1.18	2.35	0.13	0.16	0.02	1.66
Physical activity (good)	0.76	0.29	0.59	0.66	0.15	2.95
Marital status		0.66	0.72			
Single	1.76	0.23	0.63	0.43	0.01	13.44
Married	1.33	0.64	0.43	0.35	0.03	4.69
Family history	0.53	0.86	0.35	1.63	0.58	4.58
HDL		2.014	0.365			
HDL (male: <0.9 mmol/L)	0.57	1.42	0.23	1.97	0.65	5.98
HDL (female: <1.15 mmol/L)	0.78	1.52	0.22	2.62	0.57	12.05
LDL (>2.6 mmol/L)	0.71	0.12	0.73	0.78	0.19	3.13
TC (≥5.2 mmol/L)	0.81	0.12	0.73	0.75	0.15	3.69
FBG (>6.61 mmol/L)	0.60	3.74	0.05*	3.21	0.98	10.47
HbA1c (>6.5%)	0.58	2.23	0.14	0.42	0.14	1.31
C-peptide (>3.1 ng/mL)	1.40	2.23	0.14	0.12	0.01	1.93
Constant	3.04	1.30	0.25	0.03		

Reference category. OR = odds ratio. S.E = standard error. CI = confidence interval. Association significant was set at *p* < 0.05 (*) level (2-tailed). Multiple logistic regression was adopted in this analysis.

**Table 11:** Heat maps of the bivariate analysis for the correlation study determined using Spearman’s correlation coefficient.

Variables	Non-DM				DM			
	Vitamin D_3_	Calcium	α-tocopherol	Total Vitamin E	Vitamin D_3_	Calcium	α-tocopherol	Total Vitamin E
Ethnicity	0.24	0.08	−0.14	−0.17	0.09	0.10	−0.04	−0.13
Age	0.04	0.12	0.24	0.30 *	0.06	0.05	−0.19	0.06
Gender	0.11	0.11	−0.1	−0.11	0.09	0.10	−0.04	−0.13
Family history of diabetes	0.03	0.16	−0.17	−0.07	0.03	0.40 **	0.03	−0.05
Smoking status	0	0	−0.06	−0.04	−0.03	0.37 **	0.24	0.41 **
Physical activity	0.23	0.02	0.19	0.17	−0.02	0.09	0.12	−0.11
Waist circumference (cm)	0.08	−0.09	0.40 **	0.27	−0.12	0.39 **	0.03	−0.07
BMI (kg.m^−2^)	0.03	−0.18	0.19	0.19	0.11	−0.03	−0.11	−0.25
HbA1c (%)	−0.03	−0.24	0.05	0.22	−0.05	0.07	0.38 **	0.46 **
FBG (mmol/L)	−0.25	0.22	−0.21	−0.22	−0.02	0.11	−0.01	0.07
C-Peptide (ng/mL)	−0.11	0.35 *	−0.2	−0.14	0.05	0.02	0.14	0.08
HDL-C (mmol/L)	0.15	−0.34 *	0.23	0.16	0.30 *	−0.36 *	−0.05	−0.14
LDL-C (mmol/L)	0.19	−0.23	0.21	0.24	0.12	−0.32 *	0.03	0.04
TG (mmol/L)	0.03	−0.08	0.09	0.16	0.09	−0.37 **	0.14	0.27
TC (mmol/L)	0.14	−0.09	0.07	0.19	0.03	−0.39 **	0.04	0.02
Vitamin D_3_ (ng/mL)	1	−0.08	0.07	−0.09	1	−0.12	0.03	0.06
Calcium (mg/dL)	−0.08	1	−0.08	−0.14	−0.12	1	0.10	0.1
α-tocopherol (μg/mL)	0.07	−0.08	1	0.72 **	0.03	0.1	1	0.69 **
Total vitamin E (μg/mL)	0.08	−0.34 *	0.72 **	1	−0.05	0.27	0.69 **	1

Colour blue indicates a negative correlation, whereas red shows a positive correlation. The asterisk (*) and (**) indicate statistical significance at *p* < 0.05 and *p* < 0.001, respectively.
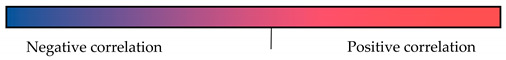

## Data Availability

Data can be made available on request.

## Supplementary Materials

The following are available online at https://www.mdpi.com/article/10.3390/nu13072288/s1, File S1: A questionnaire template that was filled by the subjects.
