# Supplementary material for: Correlation between Levels of Vitamins D3 and E in Type 2 Diabetes Mellitus: A Case-Control Study in Serdang, Selangor, Malaysia
Source: Nutrients. 2021 Jul 1;13(7):2288. doi: 10.3390/nu13072288 (PMC8308395; doi:10.3390/nu13072288)
Supplement: Supplementary file 1 [file nutrients-13-02288-s001.zip › nutrients-1249224-supplementary.pdf]

BORANG SOAL SELIDIK/ *QUESTIONNAIRE FORM*

This questionaire will be conducted as an interview.

1. Nama / *Name*:

2. No kad pengenalan (baru) / *New IC number*:

3. Tarikh lahir / *Date of birth*:

4. Umur / *Age*:

5. Alamat rumah / *Home address*:

6. Alamat kerja / *Workplace address*:

7. No telefon / *Phone number*:

8. Bangsa / *Ethnicity*:

9. Agama / *Religion* :

10. Taraf pendidikan paling tinggi / *Highest education level*:

    a. Tidak bersekolah / *Never schooled*
    b. Sekolah rendah / *Primary school*
    c. Institusi pengajian tingi / *Secondary school*
    d. Lain-lain , nyatakan / *Others , specify* : ______________________
    e. Jumlah tahun pendidikan / *Total years of education* : ______________________

11. Status perkahwinan / *Marital status*:

    a. Bujang / *Single*
    b. Berkahwin / *Married*
    c. Bercerai / *Divorced* (Sudah berapa lama menjadi duda? / *For how long have you been a divorcee*? Tahun/*Year* __________

12. Adakah anda tinggal........... / *Are you living*............?

    a. Sendirian / *Alone*
    b. Dengan isteri / *With wife*
    c. Dengan isteri dan / *With wife & children*
    d. Dengan / *With children*
    e. Lain-lain , nyatakan / *Others , specify* ______________________

13. Apakan status pekerjaan anda sekarang? / *What is your current job status*? ________________

14. Berapakah anggaran atau purata pendapatan jumlah pendapatan isi rumah? / *How much is the average monthly income for the whole family*? RM ________________

15. Adakah ibubapa, adik beradik atau saudara mara terdekat anda yang menghidap diabetes? / *Are there any of your parents, siblings or close relative been diagnosed with diabetes*?

____

16. Pernahkah anda menggunakan hasil tembakau/merokok? (Walaupun sekali guna) / *Have you ever used any tobacco products/smoking? (Even once)*

a. Ya / *Yes*

   Jika ya nytakan ________ batang per hari / *If yes specify* ________ *sticks per day*

b. Tidak , *No*
c. Bekas perokok (berapa tahun telah berhenti _______ jika kurang daripada satu tahun , nyatakan berapa batang rokok sehari _____)

Ex- smoker. (How long have you quit smoking ______years, if less than one year, how many stick a day ________ *)*

17. Pernahkah anda mengambil alcohol? (Walaupun sekali guna) / *Have you ever taken alcohol drink? (Even once).*

a. Ya / *Yes*
   Jika ya nyatakan_______ gelas per hari / perminggu / perbulan / *If yes, specify* _______ *glass per day / per week /per month*
b. Tidak / *No*
c. Bekas peminum alcohol (berapa tahun telah berhenti _______ jika , kurang daripada satu tahun , nyatakan berapa gelas alcohol sehari _______)

Ex alcohol (How long have you quit alcohol _____ *years, If less than one year, how many glass a day* ______ *)*

d. EJ / RA

18. Adakah anda menghidapi sebarang penyakit kronik berikut? / *Do you have any chronic diseases other than diabetes?*

a. Sakit jantung / *Heart dieases*

Ya / *Yes* 2. Tidak / *No* 3. Tidak fohu / Don't Know

b. Apakah Anda menderita setiap jenis masalah pencernaan/*gastrointestinal problem*

Ya / *Yes* 2. Tidak / *No* 3. Tidak fohu / Don't Know

c. *Hyperlipidemia*

Ya / *Yes* 2. Tidak / *No* 3. Tidak fohu / Don't Know

d. *Renaldisease*

Ya / *Yes* 2. Tidak / *No* 3. Tidak fohu / Don't Know

e. Arthrities / *Arthrities*

Ya / *Yes* 2. Tidak / *No* 3. Tidak fohu / Don't Know

f. Epilepsi / *Epilepsy*

Ya / *Yes* 2. Tidak / *No* 3. Tidak fohu / Don't Know

g. Lain-lain , sila nyatakan / *Others , specify* : ________________

19.Berapa lamakah anda menghidapi kencing manis/*How long have you had diabetes*? . . . . .

20. Senaraikan sebarang ubat-ubatan atau makanan tambahan atau herba yang sedang anda ambil /*List any medications or supplements or herbs you are currently taking.*

a. Nama /*Name* . . . .

b. Dos/*Dose*. . . . .

c. Masa Diambil/*Time Taken*. . . . .

21. Jika anda mengambil insulin: (sila bulatka) Adakah anda menyuntik insulin dengan /*If you take insulin: (please circle)Do you inject insulin with:*
a. picagari /*a syringe*
b. pen insulin /*an insulin pen*
c. pam insulin/*an insulin pump*
22. Pernahkah anda terlupa untuk mengambil ubat kencing manis anda /*Have you ever forgotten to take your diabetes medication?* . . . .
Jika ya , apa yang anda lakukan? *If yes, what did you do?* . . . .

23. Adakah anda memantau paras gula dalam darah anda/*Do you monitor your blood sugar?* . . . .

Jika ya , berapa kali anda memantau setiap hari/*If yes, how many times do you monitor per day?*

a. Keputusan biasa:*Usual results:*
b. Puasa */Fasting* _______
c. Sebelum makan */Before meals* ______
d. 2 jam selepas makan */2 hours after meals* _______
e. Tidur/ *Bedtime* _______

24. Adakah anda pernah diberikan rancangan pemakanan /*Have you ever been given a meal plan?* . . . .

25. Berapa kali anda makan setiap hari /*How many times do you eat per day?*
No Kekerapan makan /*No. of meals* ___________ Bilangan makanan ringan /*No. of snacks* ____________

26. Berapa kali seminggu anda makan di luar rumah /*How many times a week do you eat away from home?* . . . .

27. Bagaimana perasaan atau tekanan anda mempengaruhi cara pemakanan anda /*How does your mood or stress affect your eating?* . . . .

28. Penggunaan makanan segera / Makan dengan cepat*/ Use convenience foods Eat rapidly*
Senaraikan sebarang alahan makanan / intoleran /*List any food allergies / intolerances:* . . . .

29. Adakah anda mempunyai apa-apa keperluan diet khas atau amalan agama /*Do you have any special dietary needs or religious observances?*
Jika ya , sila jelaskan /*If yes, please explain:* . . . .

30. Adakah terdapat banyak tekanan dalam hidup anda/*Is there much stress in your life?*
Jika ya , terangkan *If yes, explain:* . . . .

31. Adakah anda aktif secara fizikal /*Are you physically active?* . . . .
Jika ya , jenis (s) aktiviti fizikal / *If yes, type(s) of physical activity:* . . . .
Berapa kerap/*How often?* ____________________
Berapa lama/ *How long?* ______________________

32. Bagaimana anda menggambarkan kesihatan anda/*How would you describe your general health?*
Cemerlang /*Excellent*.....Baik/*Good* ….. Sederhana/*Fair*..…Lemah/ *Poor*…..

33. Bagaimana anda menilai pemahaman anda tentang diabetes/*How would you rate your understanding of diabetes?*
Cemerlang /*Excellent*.....Baik/*Good* ….. Sederhana/*Fair*..…Lemah/ *Poor*…..

34. Bagaimana perasaan anda tentang menghidapi diabetes/*How do you feel about having diabetes?*
. . . .
